# Serological and Molecular Detection of Bovine Brucellosis at Institutional Livestock Farms in Punjab, Pakistan

**DOI:** 10.3390/ijerph17041412

**Published:** 2020-02-21

**Authors:** Tariq Jamil, Falk Melzer, Muhammad Saqib, Asim Shahzad, Khushal Khan Kasi, Muhammad Hammad Hussain, Imaad Rashid, Usman Tahir, Iahtasham Khan, Muhammad Haleem Tayyab, Sami Ullah, Mashkoor Mohsin, Muhammad Khalid Mansoor, Stefan Schwarz, Heinrich Neubauer

**Affiliations:** 1 Institute of Bacterial Infections and Zoonoses, Friedrich-Loeffler-Institut, 07743 Jena, Germany; falk.melzer@fli.de (F.M.); heinrich.neubauer@fli.de (H.N.); 2 Institute of Microbiology and Epizootics, Freie Universität Berlin, 14163 Berlin, Germany; stefan.schwarz@fu-berlin.de; 3 Department of Clinical Medicine and Surgery, Faculty of Veterinary Science, University of Agriculture, Faisalabad 38000, Pakistan; drsaqib_vet@hotmail.com (M.S.); imaad.rasheed@gmail.com (I.R.); drmhkhan381@gmail.com (M.H.T.); sami.ullah@gmail.com (S.U.); 4 Department of Pathology, Faculty of Veterinary Science, University of Agriculture, Faisalabad 38000, Pakistan; dr.asimshahzad@gmail.com; 5 Disease Investigation Laboratory, Livestock and Dairy Development Department, Government of Baluchistan, Quetta 87300, Pakistan; khushal.kasi@fli.de; 6 Institute of Epidemiology, Friedrich-Loeffler-Institut, 17493 Greifswald-Insel Riems, Germany; 7 1 Vance Street, 2565 Bardia, New South Wales, Australia; m.hammad.hussain@gmail.com; 8 Livestock and Dairy Development, Government of Punjab, Lahore 54100, Pakistan; usmantahir2006@gmail.com; 9 Section of Epidemiology and Public Health, University of Veterinary and Animal Sciences, Lahore, sub-campus Jhang, 12-Km Chiniot Road, Jhang 35200, Pakistan; iahtasham.khan@uvas.edu.pk; 10 Institute of Microbiology, University of Agriculture, Faisalabad 38000, Pakistan; mashkoormohsin@uaf.edu.pk; 11 Department of Microbiology, Faculty of Veterinary Science, Cholistan University of Veterinary and Animal Sciences, Bahawalpur 63100, Pakistan; mkhalidmansoor@cuvas.edu.pk

**Keywords:** bovine brucellosis, zoonosis, *Brucella abortus*, Pakistan

## Abstract

Bovine brucellosis remains a persistent infection in ruminants in Pakistan. A total of 828 (409 buffaloes and 419 cattle) sera were collected from 11 institutional-owned livestock farms in Punjab, Pakistan. The samples were tested by rose bengal plate agglutination test (RBPT) and indirect enzyme-linked immunosorbent assay (iELISA). The seroprevalence along with 95% confidence interval (CI) was determined. Univariable and multivariable analysis of the epidemiological background data was conducted and odds ratio (OR) was calculated to understand any association between the risk factors and the seroprevalence. An overall seroprevalence of 3.9% (Positive/Tested = 32/828) and 3.3% (27/828) was detected by RBPT and iELISA, respectively. The seroprevalence of 5.6% (CI 3.6–8.3) and 4.7%, (CI 2.8–7.2) and the odds ratio of 2.63 (CI 1.20–5.77) and 2.50 (CI 1.08–5.78) for testing positive by RBPT and iELISA, respectively were significantly higher (*p* < 0.05) in buffaloes than in cattle. Breed, sex, history of abortion and retention of fetal membranes (RFM) in the animals were not found statistically significantly associated with the infection. RBPT and iELISA based results agreed almost perfect (*k* = 0.877). In total, *Brucella abortus*-DNA (9/27) was amplified from seropositive samples by real-time polymerase chain reaction. This study identified for the first time the etiological agents of brucellosis at a molecular level at institutional-owned livestock farms in Pakistan.

## 1. Introduction

Brucellosis is a bacterial zoonosis caused by bacteria of the genus *Brucella* (*B.*). They are non-spore forming, non-motile, non-hemolytic and facultative intra-cellular living, Gram-negative coccobacilli. Although Brucellae show a certain host preference, e.g., *B. abortus* prefers bovines and *B. melitensis* small ruminants, cross-species transmission does occur when different animals are in close contact with each other [1,2,3,4,5,6]. Brucellosis occurs worldwide, especially in developing and tropical countries, whereas North and Central Europe, Australia, New Zealand, Japan, and Canada are considered as being free from conventional brucellosis in domestic animals [7]. Abortion in the last trimester and retention of fetal membranes (RFM) are the characteristic signs in female animals whilst orchitis and epididymitis commonly occur in males however, the infection may stay asymptomatic and the infected animals may remain undiagnosed [8]. Infected animals shed the bacteria through vaginal and milk secretions in the environment [9]. Brucellosis is usually transmitted in animals either by direct contact or through ingestion of contaminated feed or water whereas in humans, it mainly occurs through ingestion of contaminated milk [10,11]. Humans are accidental hosts for this infection and could be prevented by eliminating the infection in animals that often have close contact with humans [12,13].

The diagnostic confirmation depends on the clinical history, laboratory-based examination of biological specimens, e.g., serum and milk and upon the situation of the disease in the area. The serological examination includes rose bengal plate test (RBPT), enzyme-linked immunosorbent assay (ELISA), serum agglutination test (SAT), complement fixation test (CFT) and milk ring test (MRT) followed by molecular biological investigation, e.g., polymerase chain reaction (PCR), isolation, biochemical identification and molecular typing e.g., multilocus sequence typing (MLST), single nucleotide polymorphism (SNP) and multiple locus variable number tandem repeat analysis (MLVA) etc. [14,15]. Vaccination and treatment of brucellosis in farm animals are not considered 100% safe for human health, hence are forbidden in many countries [7,16,17,18,19].

Pakistan is an agriculture-based country where livestock plays an integral role in the agriculture economy. More than 8.0 million families are associated with livestock raising and derive ≥35% of their income from livestock production in the country [20]. Brucellosis is considered an endemic infection in the ruminants in Pakistan [21]. Bovines are the primary source of milk in the country, and for milk production, Pakistan has been among the top countries in the world [22]. Our aim for this study was to estimate the burden of brucellosis in buffaloes and cattle reared at 11 institutional-owned livestock farms by serology and detect the etiology by molecular biology. To the best of our knowledge, this is the first study to identify brucellosis at molecular level at these farms in Pakistan.

## 2. Materials and Methods 

**Figure 1:**
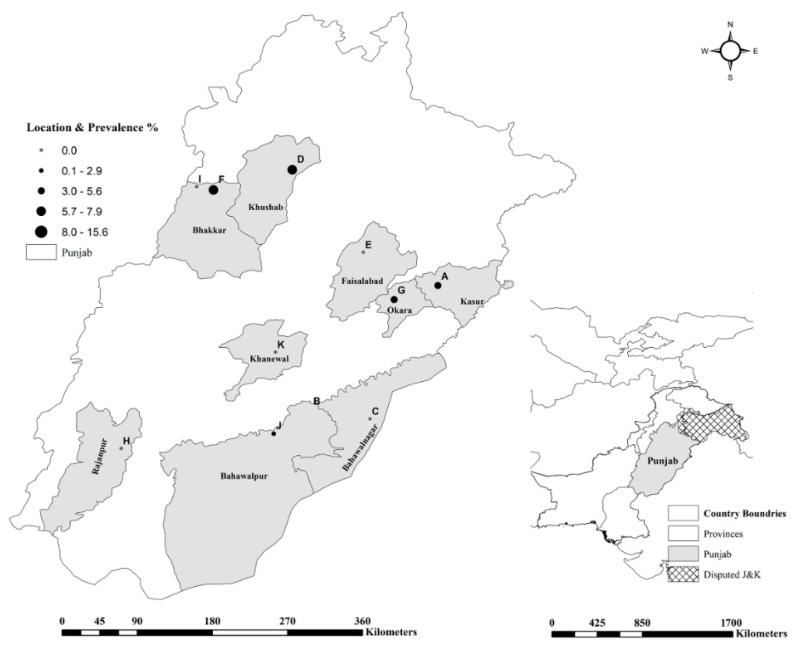
Geographic distribution of brucellosis infection among livestock farms in Punjab, Pakistan.

For this study, 11 institutional livestock farms (Farms A–K), administered by the Livestock and Dairy Development (L&DD), Government of Punjab, Lahore and University of Agriculture (UAF), Faisalabad, representing different geographical locations (Figure 1) were selected as described previously [23,24]. Since the prevalence of brucellosis was considered unknown at these selected farms, the sampling frame was constructed to investigate brucellosis at expected prevalence of 50%, 95% confidence interval (CI) and 5% desired absolute precision [25]. This required that at least 385 samples from buffaloes and cattle each to be tested from the selected farms. This sample size was further divided according to the population proportion of these animals at each farm. A total of 828 sera (409 buffalo and 419 cattle) were sampled. Animals were randomly selected and properly restrained before the blood was drawn into a 9-mL vacutainer tube by puncturing the jugular vein. Samples were labelled with the animal identification information (tag number, age, breed, and sex). Epidemiological information regarding the animal and herd level variables were recorded on a questionnaire. The samples were then transported to the Department of Clinical Medicine and Surgery, Faculty of Veterinary Science, University of Agriculture, Faisalabad, Pakistan where serum was separated and stored at −20 °C until further testing.

Sera were screened for brucellosis by RBPT using Pourquier^®^ Rose Bengal Antigen (IDEXX, Montpellier, France) by using bovine bacterial positive and negative control sera provided by Friedrich-Loeffler-Institut (FLI), Jena, Germany. It was followed by indirect-Enzyme Linked Immunosorbent Assay (iELISA) via ID Screen^®^ Brucellosis Serum Indirect Multi-species (IDVet, Grabels, France) for detection of anti-smooth-Lipopolysaccharide (LPS) antibodies (*B. abortus*, *B. melitensis* and *B. suis*) as per manufacturer’s recommendations. The sera were then subjected to DNA extraction by Blood Genomic DNA Extraction Mini Kit (Favorgen^®^, Ping-Tung, Taiwan) followed by detection/differentiation of Brucellae at species level by real-time PCR using SYBR^®^ Green as described earlier by using previously described sets of primers [26,27]. Each DNA extraction procedure was run along with *E. coli* negative controls and *B. abortus* (Veterinary Research Institute, Lahore, Pakistan) and *B. melitensis* (University of Agriculture, Faisalabad, Pakistan) [6] were used as positive controls in PCR procedure. As no reports on *B. suis* were available in the country, we considered *B. suis* was not prevalent in the area, hence no controls were used. Based on our in-house experience, a cycle threshold (Ct) value of ≤35 was considered as positive [27].

### Statistical Analysis

The statistical analysis was conducted by using the R and R-Studio software (RStudio Inc., Boston, MA, USA) [28], and maps were built using ArcGIS version 10.5.1 (ESRI, Redlands, CA, USA). The confidence interval (CI) for the proportions was estimated by the exact 95% Clopper and Pearson interval method using the binom package (binom.test function). Univariate and multivariate analysis were conducted to determine the association and risk (Odds ratio; OR) of the biologically plausible factors with the prevalence of brucellosis. The confirmation of brucellosis was considered as an outcome or dependent variable while possible risk factors were considered as explanatory or independent variables. For the independent variables, biologically plausible variables were considered. The *p* < 0.05 was considered as a level of significance. The Nagelkerke R^2^ (NR^2^) and Hosmer and Lemeshow Test (HLT) were used to evaluate the final-model fitness. An inter-rater reliability analysis using the Kappa statistics was performed to determine the agreement among two tests, i.e., RBPT and iELISA.

## 3. Results

An overall 3.9% (Positive/Tested = 32/828) and 3.3% (27/828) seroprevalence was found by RBPT and iELISA, respectively, among the livestock farms sampled in Punjab, Pakistan (Table 1 and Table 2).

For risk factor variables, the sampled animal population (*n* = 828) was divided into two categories, i.e., buffalo (*n* = 409) and cattle (*n* = 419). For the breed variable, two groups were categorized, i.e., local bred animals encompassing Nili-Ravi (*n* = 409) in buffaloes (*n* = 409) and Sahiwal (*n* = 335), Cholistani (*n* = 46) and crossbred (*n* = 38) in cattle. Based on sex, animals were grouped into buffalo males (*n* = 6) and females (*n* = 403) and cattle males (*n* = 43) and females (*n* = 376). Age groups, i.e., <2 years comprised young stock in buffaloes (*n* = 77) and cattle (*n* = 95) and ≥2 years comprised bulls, heifers, pregnant and lactating animals in buffaloes (*n* = 332) and cattle (*n* = 324). Although retention of fetal membranes (RFM) and history of abortion are purely related to females and prior pregnancy status, males and heifers were considered animals being negative for prior history for RFM and abortion. All sampled animals (*n* = 828) had no prior history of vaccination against brucellosis at these farms. At the time of sampling, the 11 farms had either only buffaloes (*n* = 4), only cattle (*n* = 4) or both, buffaloes and cattle (*n* = 3) (Table 1).

Species wise in buffaloes, the mean seroprevalence was 5.62% (23/409; range 0–18.75%) by RBPT and 4.64% (19/409; range 0–15.62%) by iELISA at the sampled farms. The highest seroprevalence was found at Farm B with gradual decrease to 0% at Farm C and Farm E respectively, by both tests. Similarly in cattle, the mean seroprevalence was 2.15% (9/419; range 0–6.3%) by RBPT and 1.91% (8/419; range 0–5.52%) by iELISA with highest at Farm G decreasing to 0% at Farms E, F, H, I and K by both tests. The seroprevalence varied statistically significant (*p* < 0.05) by both RBPT (Chi-square value; χ^2^ = 6.729) and iELISA (χ^2^ = 4.69) between buffaloes and cattle at eleven farms (Table 1). The mean RBPT-based seroprevalence (3.9%) varied (0–18.8%) statistically significant (χ^2^ = 39.680, *p* < 0.05) among the sampled livestock farms. A similar pattern was found for the iELISA-based seroprevalence (3.3%) varying (0–15.6%) statistically significant (χ^2^ = 33.498, *p* < 0.05) among the sampled farms (Table 2). 

In univariate analysis, farm-related variables e.g., feeding methods, herd type, breeding methods and farm environment did not show statistically significant associations (*p* > 0.05) to the seropositivity for brucellosis in both buffaloes and cattle. In animal related variables, species of the animals (buffalo or cattle) did show statistically significant association (*p* < 0.05) with odds ratio of 2.7 (1.24–5.94; 95% CI) in buffalo with reference to cattle. Breed of the animal (local breed or cross-breed) and sex of the animal (male or female) could not be determined whereas, age groups (<2 years and ≥2 years), tick infestation, RFM and history of abortion were not found statistically significantly associated. However, age grouping showed a closer value to the significance level (Table 3). Multivariate analysis for species differences showed a statistically significant association (*p* < 0.05) with an Odds ratio of 2.63 (1.20–5.77; 95% CI) in buffaloes as compared to the cattle. Age group difference did not show a statistically significant association, however, and was found closer (*p* = 0.065) to the level of significance (Table 4). 

Samples from Farm C, Farm E, Farm H, Farm I, and Farm K did not show any positive by serology hence were not subjected for DNA extraction and molecular detection of *Brucella*-DNA. Out of total, 27 seropositive samples, 9 samples (6 buffaloes and 3 cattle) did amplify *Brucella*-DNA by conventional and subsequently *B. abortus*-DNA by real-time PCR.

In total, 828 serum samples were tested through RBPT and iELISA. Out of these, 32 samples were found positive in RBPT and 27 in iELISA (Table 2). Out of the 32 RBPT positive samples, 26 were iELISA positive also. (Table 5). The agreement between RBPT and iELISA results was found almost perfect (*k* = 0.877) (Table 6).

## 4. Discussion

Serology remains an important tool in brucellosis diagnosis and RBPT and iELISA were used for screening of bovine sera in this study. RBPT has been widely accepted as a test with higher sensitivity and lower specificity as it can potentially cross-react with antibodies to other non-*Brucella* antigens [29]. Meanwhile, the iELISA is considered to be sensitive and could be used as a single diagnostic criterion at standardized labs [30]. However, RBPT remains an adequate screening test based upon the disease epidemiology, purpose of the diagnostic criteria and availability of the resources [31,32]. Therefore, we tested our sera by both tests and determined the possible agreement between these two tests (Table 5 and Table 6). These serological tests do not differentiate between the *Brucella* species as *B. abortus*, *B. melitensis* and *B. suis* share common antigenic LPS. DNA-based tests, e.g., PCR, are able to differentiate at species level with high specificity. Clinical samples (e.g., serum and milk) contain lower amounts of bacterial DNA hence the sensitivity of PCR becomes really low. As the amount of bacterial DNA may depend upon the stage of the infection e.g., in chronic cases it is very unlikely to detect *Brucella*-DNA in serum samples. Real-time PCR provides a robust diagnostic solution with higher sensitivity, but also requires higher costs for the performance of this test. Isolation of Brucellae remains the gold standard for brucellosis diagnosis, but is less efficient, laborious and requires advanced laboratory conditions, e.g., level 3 biosecurity laboratories. A SYBR^®^ Green-based assay was thus used for confirmation and differentiation of the etiology at species level. 

**Figure 2:**
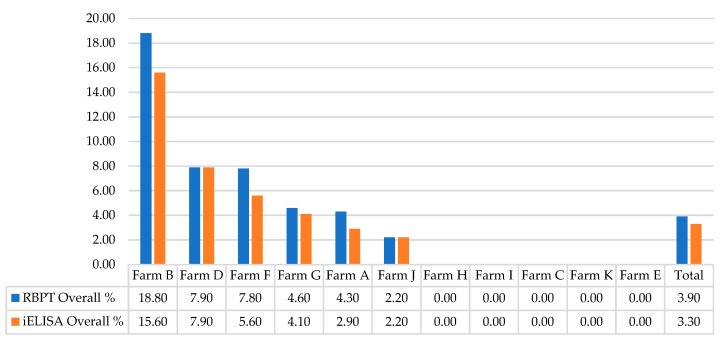
Farm-wise seroprevalence of brucellosis. RBPT—Rose Bengal Plate Agglutination Test; iELISA—Indirect Enzyme-Linked Immunosorbent Assay.

In comparison to RBPT, iELISA and other diagnostic tests, similar results, as obtained in our study, were found previously in Pakistan [33]. However, statistically significant (*p* < 0.05) lower seroprevalence rates were detected by RBPT compared to iELISA [21]. This variability might be due to the difference in number and infection status of sampled animals, consumables used, laboratory conditions and personal expertise. Among the sampled farms, RBPT- and iELISA-based seroprevalence differed statistically significant (*p* < 0.05) ranging 0–18.8% and 0–15.6%, respectively. Although iELISA-based seroprevalence was found to be slightly lower than that of RBPT, the seroprevalence trend was the same at the farms for both RBPT and iELISA (Table 2). Highest seroprevalence 18.8% and 15.6% was found at Farm B followed by Farm D, Farm F, Farm G, Farm A, and Farm J by RBPT and iELISA, respectively (Figure 2).

The seroprevalence pattern for buffaloes and cattle based on the location of the farms varied statistically significant (*p* < 0.05) (Table 1). Farm B, Farm F, Farm D, and Farm A had seropositive buffaloes whereas only Farm G and Farm J had seropositive cattle. Herd size, farm management practices, and contact with other domestic animals have been associated with the infection occurrence at different farms/herds [3,29,34,35]. However, the results are contradicted [36,37,38] and remain undetermined elsewhere in the country [39,40].

A variability in seroprevalence has been observed at institutional-owned, private-owned, general livestock population and rural animal holdings in Pakistan previously, based on these tests [21,35,36,41,42]. Brucellosis is an established professional health hazard in Pakistan [11,43,44,45]. Both *B. abortus* and *B. melitensis* have been identified [2,3,4,5,46,47,48]. Despite a great influx of brucellosis reporting in the recent past, livestock holders seem to be unaware of the infection [35]. Brucellosis is frequently reported at intensive dairy farms as compared to small animal holders in the country [29]. At the farms level, institutional-owned livestock farms tend to be less susceptible to the infection, maybe because of better screening, culling, hygiene and veterinary health facilitation programs than private livestock farms and a statistically significant difference (*p* < 0.05) has been reported [21,34,39,41], however disagreement does exist [42]. One of the major causes of brucellosis outbreaks especially at private-owned farms is the breach in biosecurity, i.e., the introduction of carrier animals (i.e., most often subclinical infected animals) into the existing herd without prior screening [4,49]. The infection remains unsuspected until abortion storm occurs and/or animals are screened for brucellosis. Brucellae do respond well to most of the commercially available antimicrobial agents, routine disinfectants and sterilization techniques although hints of resistance are reported [50,51]. They are killed by UV/sunlight exposure, 70% ethyl alcohol and by autoclavation [52,53]. Animals often conceive subsequently but remain carriers for their life. Veterinarians, municipal workers, butchers, technicians and householders acquire the infection unintentionally during unprotected handling of the infected animals [12,54].

More seropositive samples were found among the buffaloes i.e., 5.6% (23/409) and 4.7% (19/407) by iELISA than among cattle 2.2% (9/419) and 1.9% (8/419) by RBPT and iELISA, respectively, and that was statistically significant (Table 2). This difference is further clarified by multivariate analysis where buffaloes depict higher risk odds ratios than cattle for the infection (Table 4). Similar statistically significant results have been reported previously [21,36,55] however, contradictive results by Seed et al. [3] and without statistical determination are also reported [21,40]. To the best of our understanding, the real reason for biological affinity of buffaloes towards brucellosis remains unclear.

Although our study could not find statistically significant association for breed of the animals with brucellosis, the crossbred and exotic cattle have been previously reported to be more prone to the infection as compared to the local/indigenous breeds [49,56,57,58,59]. Specifically, within the cattle, breeds, i.e., Sahiwal, Cholistani, and crossbred, univariate analysis did not show statistical significance (*p* > 0.05) with the infection (Table 3). This might be due to the difference in geography or sampling bias because of the presence of a higher number of local/indigenous cattle population at these farms. Nevertheless, *Nramp1* gene is associated with brucellosis resistance [60,61,62].

Our study found only females positive for brucellosis and could not determine a statistical association, although sex of the animals was not associated statistically significant (*p* > 0.05) in previous reports [21,33,41] although associated by Ali et al. [36]. This may be due the fact that relatively fewer bulls are kept at dairy purpose farms because of increasing local artificial insemination facilities and interest of the farm owners in female animals for production [21]. However, controversial arguments do exist [63].

More animals were tested positive in age group ≥ 2 years but were found statistically non-significant (*p* > 0.05) to the infection as supported by the previous findings [3,33,36,41]. Similar results are reported in cattle but a statistically significant association was found in buffaloes [55]. A similar trend was observed with the increase in age, but statistical significance was not determined [40]. However, mature animals remain at higher risks [36]. Young animals contract the infection when fed on contaminated colostrum or milk from infected dames. Although our study analyzed the relation of presence of ticks with brucellosis, a statistically non-significant relation was found. Similarly, the multivariate analysis did not show any statistically significant association (*p* > 0.05) (Table 4). External parasites and ticks have not been related to brucellosis epidemiology so far [52].

RFM and history of abortion did not show statistically significant association (*p* > 0.05) to the infection in our study, maybe because of the better health and husbandry services at these farms. However, this observation has been contradicted by previous reports that have found a significant association [3,33,36,38,55].

## 5. Conclusions

Brucellosis remains a persistent infection in bovines in Pakistan. Husbandry practices might play a role determining the occurrence of the infection at a specific farm/location. Buffaloes seem to be at higher risk when compared to cattle. Although, specific breed, sex of the animals, age and history of reproductive disorders could not be associated in the study, based on previous literature, these factors should not be ignored while screening for brucellosis. *B. abortus* was detected to be the cause of infection. Small ruminants as well as non-preferred hosts (dogs, equines, etc.) in close contact are needed to be tested to determine the presence/transmission role of these animals to the infection. A standardization of the diagnostic system, e.g., ELISA and PCR, is recommended. Routine diagnostic screening, culling, biosafety, biosecurity, and quarantine measures are needed to continue especially when introducing new animals to the existing herd. The milk chain is needed to be traceable at these farms to avoid unintentional mixing of contaminated/antimicrobial-treated milk into the main supply chain to avoid human transmission. The pasteurization of milk would be highly recommended. Proper disinfection and sterilization of the area and personal protection is needed in case of abortion outbreaks at farms. Isolation and identification of the etiological agents at molecular level is recommended when required facilities are available. Based on the results in this study, RBPT can be used sufficiently for the purpose of screening for brucellosis in farm animals under local conditions. This study is the first in which *Brucella* was identified to the species level at organized institutional livestock farms in Pakistan.

## Figures and Tables

**Table 1:** Seroprevalence in cattle and buffaloes sampled from various farms.

Sr. No.	Farm Name	Buffalo				Cattle				Real-Time PCR (SYBR^®^ Green)		
		RBPT		iELISA		RBPT		iELISA				
		Pos./Tested	Prev.% (95% CI)	Pos./Tested	Prev.% (95% CI)	Pos./Tested	Prev.% (95% CI)	Pos./Tested	Prev.% (95% CI)	Buffalo	Cow	Total
1	Farm A	3/70	4.3 (0.9–12)	2/70	2.9 (0.3–9.9)	-	-	-	-	0	0	0
2	Farm B	6/32	18.8 (7.2–36.4)	5/32	15.6 (5.3–32.8)	-	-	-	-	3	0	3
3	Farm C	0/35	0 (0–10)	0/35	0 (0–10)	-	-	-	-	0	0	0
4	Farm D	6/76	7.9 (3–16.4)	6/76	7.9 (3–16.4)	-	-	-	-	2	0	2
5	Farm E	0/58	0 (0–6.2)	0/58	0 (0–6.2)	0/45	0 (0–7.9)	0/45	0 (0–7.9)	0	0	0
6	Farm F	7/71	9.9 (4.1–19.3)	5/71	7 (2.3–15.7)	0/19	0 (0–17.6)	0/19	0 (0–17.6)	1	0	1
7	Farm G	1/67	1.5 (0–8)	1/67	1.5 (0–8)	8/127	6.3 (2.8–12)	7/127	5.5 (2.2–11)	0	3	3
8	Farm H	-	-	-	-	0/23	0 (0–14.8)	0/23	0 (0–14.8)	0	0	0
9	Farm I	-	-	-	-	0/75	0 (0–4.8)	0/75	0 (0–4.8)	0	0	0
10	Farm J	-	-	-	-	1/46	2.2 (0.1–11.5)	1/46	2.2 (0.1–11.5)	0	0	0
11	Farm K	-	-	-	-	0/84	0 (0–4.3)	0/84	0 (0–4.3)	0	0	0
	**Total**	**23/409**	**5.6 (3.6–8.3)**	**19/409**	**4.7 (2.8–7.2)**	**9/419**	**2.2 (1–4)**	**8/419**	**1.9 (0.8–3.7)**	**6/19**	**3/8**	**9/27**

Sr. No.—Serial number; RBPT—Rose Bengal Plate Agglutination Test; iELISA—Indirect Enzyme-Linked Immunosorbent Assay; PCR—Polymerase Chain Reaction; Pos.—Positive; Prev.—Prevalence; CI—Confidence interval; RBPT-based seroprevalence varied significantly between cattle and buffaloes, χ^2^ = 6.729, *p* = 0.009. iELISA-based seroprevalence varied significantly between cattle and buffaloes, χ^2^ = 4.690, *p* = 0.030.

**Table 2:** Overall Seroprevalence of brucellosis in cattle and buffaloes sampled from different farms.

Farm Name	RBPT Overall		iELISA Overall	
	Pos./Tested	Prev.% (95% CI)	Pos./Tested	Prev.% (95% CI)
Farm A	3/70	4.3 (0.9–12)	2/70	2.9 (0.3–9.9)
Farm B	6/32	18.8 (7.2–36.4)	5/32	15.6 (5.3–32.8)
Farm C	0/35	0 (0–10)	0/35	0 (0–10)
Farm D	6/76	7.9 (3–16.4)	6/76	7.9 (3–16.4)
Farm E	0/103	0 (0–3.5)	0/103	0 (0–3.5)
Farm F	7/90	7.8 (3.2–15.4)	5/90	5.6 (1.8–12.5)
Farm G	9/194	4.6 (2.1–8.6)	8/194	4.1 (1.8–8)
Farm H	0/23	0 (0–14.8)	0/23	0 (0–14.8)
Farm I	0/75	0 (0–4.8)	0/75	0 (0–4.8)
Farm J	1/46	2.2 (0.1–11.5)	1/46	2.2 (0.1–11.5)
Farm K	0/84	0 (0–4.3)	0/84	0 (0–4.3)
**Total**	**32/828**	**3.9 (2.7–5.4)**	**27/828**	**3.3 (2.2–4.7)**

RBPT-based prevalence differ significantly among sampled farms, χ^2^ = 39.680, *p* < 0.001. iELISA-based prevalence differs significantly among sampled farms, χ^2^ = 33.498, *p* < 0.001.

**Table 3:** Univariable in cattle and buffaloes at animal level.

Variable	Category	Pos./Tested	Prev.% (95% CI)	Odds Ratio	95% CI	*p*-Value *
**Species**	Cattle	9/419	2.2 (1–4)	Ref	-	0.012
	Buffaloes	23/409	5.6 (3.6–8.3)	2.71	1.24–5.94	
**Breed**	Local	32/790	4.1 (2.8–5.7)	-	-	-
	Cross	0/38	0 (0–9.3)	-	-	
**Sex**	Female	32/779	4.1 (2.8–5.7)	-	-	-
	Male	0/49	0 (0–7.3)	-	-	
**Age groups**	<2 Years	2/172	1.2 (0.1–4.1)	Ref	-	0.056
	≥2 Years	30/656	4.6 (3.1–6.5)	4.07	0.96–17.22	
**Ticks infestation**	No	31/766	4.1 (2.8–5.7)	2.57	0.35–19.17	0.356
	Yes	1/62	1.6 (0–8.7)	Ref	-	
**RFM**	No	29/781	3.7 (2.5–5.3)	Ref	-	0.363
	Yes	3/47	6.4 (1.3–17.5)	1.77	0.52–6.03	
**History of abortion**	No	30/771	3.9 (2.6–5.5)	Ref	-	0.885
	Yes	2/57	3.5 (0.4–12.1)	1.11	0.26–4.78	

RFM—Retention of fetal membranes; Ref—Reference value; * *p* value ≤ 0.05 considered as significant.

**Table 4:** Multivariable analysis at animal level for cattle and buffaloes.

Variable	Exposure Variable	Comparison	Odds Ratio	95% CI	*p*-Value *
Species	Buffaloes	Cattle	2.63	1.20–5.77	0.016
Age group	≥2 years	<2 years	3.89	0.92–16.47	0.065

* *p* value ≤ 0.05 considered as significant; (Model fitness: Nagelkerke R^2^ (NR^2^) = 0.051, Hosmer and Lemeshow Test (HLT) = 1.028, *p* = 0.598).

**Table 5:** Comparison of results of RBPT and iELISA tests used to detect anti-*Brucella* antibodies in cattle and buffaloes.

RBPT		iELISA		Total
		Negative	Positive	
Negative	Count	795	6	801
	Expected Count	770	31	801
Positive	Count	1	26	27
	Expected Count	26	1	27
Total	Count	796	32	828
	Expected Count	796	32	828

**Table 6:** Agreement between RBPT and iELISA tests used for sero-diagnosis of brucellosis in cattle and buffaloes (*n* = 828).

Comparison	Observed Agreement	SE	Kappa Value	95% CI of Kappa	*p*-Value *
RBPT vs. iELISA	99.15%	0.046	0.877	0.787, 0.967	<0.01

SE—Standard error; * *p* value < 0.05 considered as significant
